# Development and evaluation of food preservation lessons for gardeners: application of the DESIGN process

**DOI:** 10.1017/S1368980023002926

**Published:** 2023-12-27

**Authors:** Alyssa W Beavers, Allison O Kennedy, Jessica P Blake, Sarah S Comstock

**Affiliations:** 1 Department of Nutrition and Food Science, Wayne State University, Detroit, MI 48201, USA; 2 Department of Food Science and Human Nutrition, Michigan State University, East Lansing, MI, USA

**Keywords:** Food preservation, Gardening, Food preservation education, DESIGN process

## Abstract

**Objective:**

This study presents the development and evaluation of food preservation lessons for gardeners.

**Design:**

Lessons were developed using the DESIGN process, a nutrition education program planning framework. This study examines the effectiveness of this curriculum at increasing knowledge of proper food preservation practices and increasing participants’ confidence in home food preservation, identifies challenges participants experienced with home food preservation and assesses the perceived influence of home food preservation on vegetable intake and aspects of food security. We used the DESIGN process developed by Contento and Koch to develop the curricula and used social cognitive theory to guide the lesson development. Lessons on three types of food preservation (freezing, water bath canning and pressure canning) were developed and presented to adult gardeners. The evaluation consisted of post-lesson surveys and a follow-up survey several months after the lessons.

**Setting:**

Mid-Michigan, USA.

**Participants:**

Adult gardeners.

**Results:**

Food preservation confidence increased following the lessons. At follow-up, 64 % of participants agreed or strongly agreed that they ate more fruit and vegetables because of preserving food, 57 % of respondents agreed or strongly agreed that they spend less money on food due to preserving, while 71 % reported being better able to provide food for themselves and their family. Lastly, 93 % reported feeling better about where their food comes from and wasting less food due to preserving.

**Conclusions:**

This study provides evidence that home food preservation may be beneficial in promoting fruit and vegetable intake and food security among gardeners.

Adequate intake of fruit and vegetables is one of the most health-promoting dietary factors, yet fruit and vegetable intake falls far short of recommendations in the USA^(1)^. Gardening is one viable strategy to improve fruit and vegetable intake: cross-sectional studies have found that gardeners consume fruits and vegetables more often than non-gardeners and gardening intervention studies have demonstrated an increase in fruit and vegetable intake after gardening^(2–6)^. Home or community gardens can generate a sizeable amount of produce. Several studies have found the dollar value of produce to be between $339 and $459 each year^(7–9)^. In many areas of the country, the garden harvest occurs primarily over only a few months of the year. During this time, gardeners often produce more than their households can eat fresh, and a sizeable amount of produce is given away^(7,9)^. Two studies found that on average 30 % of the total produce harvested was given away to friends, family or others^(7,9)^.

Another means to handle an abundance of garden produce in a short period of time is food preservation. Food preservation, such as canning or freezing, prevents food spoilage and maintains both the safety and quality of fruits and vegetables for up to a year or even longer. By preserving garden produce, gardeners could maintain more produce within their own households, potentially improving nutrition security and vegetable intake year-round. However, there are several barriers to home food preservation, especially canning. Identified barriers to home canning include lack of time, lack of confidence and concern over loss of time and money if canning is not successful ^(10)^. However, gardeners report a sense of pride in growing their vegetables and report a negative emotional response when their personal hard work and time is wasted, which may indicate a willingness to sacrifice additional time in order to preserve their hard work^(11)^.

Safety of home preserved food is dependent upon using a tested recipe from a reputable source and following directions exactly. Unsafe home canning practices, such as using an incorrect method, altering the recipe or failing to adjust for altitude, may persist in over 40 % of food-preserving households, despite the internet containing numerous science-based home food preservation resources^(12,13)^. Unsafe canning practices are worrisome due to the possibility of improperly home canned products developing *C. botulinum* spores and causing foodborne botulism, a potentially fatal illness that often results in hospital intensive care. These cases are extremely rare among home food preservers and are due to unsafe or outdated canning practices. Fear of foodborne illness may also deter home canning; thus, it is important that home food preservation be taught in a way that is encouraging and ultimately leads to gardeners preserving their garden harvest. While many home food preservation curricula exist, there is a lack of published evaluation data from these programmes. Therefore, there is little evidence that existing food preservation curricula increase safe home food preservation. Additionally, there is a lack of information regarding the potential for home food preservation education to impact vegetable consumption or food security. This study addresses these gaps by designing a social cognitive theory-based food preservation curriculum for gardeners and evaluating its effectiveness at increasing vegetable intake and improving food security. To develop the curricula, we use the DESIGN process, a systematic and interactive six-step curriculum planning tool for nutrition education. DESIGN is an acronym for Decide behaviour, Explore determinants, Select theory-based model, Indicate objectives, Generate plans and Nail down evaluation^(14,15)^. While the DESIGN process was created for nutrition education curricula, in our study, we apply this same process to create a curriculum focused on food safety and food preservation skills. Few research papers to date have presented the use of the DESIGN process in developing and evaluating education programs, and none have focused on food safety or food preservation.

## Methods

We present how we used the DESIGN process for designing and implementing this curriculum, and we also describe any modifications we made. In the results section, we present the findings of the post-lesson and outcome evaluations.

### Step D: decide behaviours

The first step of the DESIGN process, decide behaviour change goals of the intervention, instructs the nutrition educator to first identify health problems of the audience and current behaviours that contribute to the problem to solve. We did not have the opportunity to obtain information directly from the audience, therefore we relied on information collected from similar audiences to inform this step. Nutrition-related issues in a similar audience, low-income Michigan adults, were identified through a literature review. This revealed that low-income adults in Michigan have higher rates of diet-related diseases including diabetes, cancer and CVD but lower intakes of fruits and vegetables compared with higher income Michigan adults^(16)^. Through a review of the relevant literature on gardeners, we found that gardeners often have heavy yields of garden vegetables in summer months, leading to a large share of the garden harvest being given away^(7,17)^. Home food preservation would help gardeners keep more of their garden harvest within their own household. However, lack of time, knowledge and skills are barriers to preserving food. These findings informed our behaviour change goal: increase safe preservation of garden produce for year-round consumption.

### Step E: explore determinants

This step involves exploring the motivational and facilitating determinants that may influence the target audience making the behaviour change goal selected. We reviewed literature pertaining to diet and food values among gardeners, as well as literature on home food preservers. The literature on gardeners identified motivational determinants, the ‘why-to.’ We found that gardeners have a positive emotional connection to the food they grow. This includes feelings of pride, which results in a desire to not waste the produce that they invested time and resources to produce^(18–21)^. Gardeners also preferred their own produce over store-bought because they believe the vegetables they grow taste better and are fresher and healthier than the food from the grocery store^(11,19,21,22)^. They value knowing exactly how their garden produce is grown, which often means being grown organically or without pesticides^(11,19–21,23–25)^.

Examining the existing research literature on home food preservation, we identified facilitating determinants, or factors related to the ‘how-to’ of food preservation. Most of the existing literature surrounding home food preservation focused on canning practices in the USA. A survey examining canning practices among US households in 2005 found that potentially dangerous canning practices were prevalent: nearly one in three home canners surveyed reported altering recommended canning procedures and 44 % reported practicing ‘open-kettle canning,’ a procedure that is not recommended by canning experts^(13)^. Another study examined sources of home canning information among home canners. They found that ‘informal’ sources, such as family and the internet, are used more commonly than ‘formal’ sources such as Extension and canning books^(26)^. The high use of informal sources is concerning due to the risk of food safety issues when not following evidence-based canning recipes. For example, one study examined how well canned salsa recipes on food blogs adhere to United States Department of Agriculture (USDA) canning guidelines and found that few recipes met the guidelines for acidification, and many were missing other pertinent canning safety information^(27)^. These findings from previous research suggest that many home canners may lack knowledge and skills of safe home canning practices.

Very little information was found regarding the barriers and facilitators to home food preservation. We identified one such study: a survey of parents of young children found the most frequently cited barriers to home food preservation are lack of time and lack of skills or knowledge^(10)^. While not identified directly from the literature, we added self-efficacy in the ability to safety preserve food as a facilitating determinant. Self-efficacy is an important component of behaviour change. According to Contento and Koch, self-efficacy is ‘the belief that we have the power to produce desired results by our actions’^(14)^. Self-efficacy motivates people to persist in learning new skills and engage in new health behaviours, even when they experience challenges. Home food preservation requires highly specialised knowledge and skills. Without high self-efficacy for home food preservation, home food preservers may not persevere when faced with challenges.

### Step S: select theory-based model

The S portion of the DESIGN Process involves three components. The first is selecting a theory-based model for the curriculum based on the identified motivators and facilitators from section E. The theory-derived determinants serve to guide the development of the education in subsequent steps. We chose social cognitive theory as it best matched the determinants we found from step E, and it is used extensively in nutrition education and other health promotion fields. The key determinants of social cognitive theory are divided into three overarching categories, personal, behavioural and environmental, that reciprocally influence each other. For this step, we aligned the determinants from step E with specific components of social cognitive theory (Table 1).

**Table 1:** Results from steps E (explore determinants) and S (select theory) of the DESIGN process

Motivational ‘why to’ determinants	Determinant from social cognitive theory framework	Link to behaviour
Gardeners believe garden produce is healthier due to how it is grown, they have pride in and trust for their garden produce and gardeners prefer the taste and freshness of garden produce	Outcome expectations	These motivational determinants drive gardeners to want to not waste the produce they grow and to enjoy their produce year-round.
Facilitating action ‘how-to’ determinants	Determinant from theory framework	Link to behaviour
Preserving food, especially canning, requires specialised knowledge and skills, and unsafe food preservation practices are commonly used	Food and nutrition skills and knowledge	A lack of food preservation skills and knowledge may lead to following unsafe food preservation practices.
Confidence in ability to safely preserve food	Self-efficacy	A lack of confidence in the ability to safely preserve food may lead to not preserving when challenges are encountered.

Other components of the S section are to identify an educational philosophy and an approach to nutrition science content. To adapt the DESIGN process to our needs, we interpreted this step as ‘food safety and food preservation content.’ Regarding the educational philosophy, we wanted to build upon the positive attitudes and preferences that gardeners have for the food they grow, serving to motivate participants to preserve this produce for year-round use. Additionally, we determined that while it was essential to teach safe food preservation practices, we wanted to avoid causing undue food safety fears. In line with our educational philosophy, we chose to use only safe, tested food preservation information as the source content for our lessons (more details below in section ‘G’).

### Step I: indicate objectives

In section I, educational objectives based on the determinants from step E are written. These educational objectives are what you would like the participants to know, feel or be able to do immediately after the lessons and serve to guide the next step in the DESIGN process, generate lesson plans^(14)^. We developed two objectives related to the determinant self-efficacy: Increase confidence in the ability to safely preserve food and decrease food safety fears of home canning. We developed one related to the determinant food and nutrition skills and knowledge: Know safe canning or freezing practices. Lastly, we developed one objective pertaining to the determinant outcome expectations: Increase motivation to preserve food to avoid wasting garden produce and for year-round enjoyment.

### Step G: generate plans

The next step in the DESIGN process is generate educational plans for the intervention. This step starts with considering practical matters that may influence lesson implementation. Most notably, the COVID-19 pandemic was an important factor that influenced lesson planning. While there were several lesson components that were desired, such as canning demonstrations and food tastings, these were not included in the lessons to ensure safety. Due to the need for physical distancing, it was decided to perform the lessons completely in a classroom-type environment, and not include demonstrations in a kitchen that would have required participants to be close together. During the preparation phase, we tested adding a live water bath canning demonstration using a portable plug-in burner. However, the time needed to bring the water to boil was a barrier to implementation, and thus live demonstrations were not included in the lessons.

In line with our approach to nutrition science in step S, we used only evidence-based sources of food preservation information as the content for our lessons. Our primary sources for lesson content were the USDA Complete Guide to Home Canning and the National Center for Home Food Preservation website^(28,29)^ These two sources are considered the gold standard for safe, tested food preservation information. Other food preservation curricula that used these sources for their content were also reviewed to ensure all key content was included^(30–32)^.

The focus of generating the educational plans was to teach participants how to carry out home food preservation in a simple and easy to understand manner. Therefore, the lessons were heavily focused on the theory-based determinants of food preservation knowledge and skills. The other determinants we identified were incorporated into the lessons where possible, and Table 2 outlines how determinants were included in each lesson. The lessons primarily consisted of a PowerPoint supplemented with additional components as described below.

**Table 2:** Results from step G (generate educational plans) of the DESIGN process

Determinant	Freezing lesson	Water bath canning lesson	Pressure canning lesson
Outcome expectations/positive self-evaluation	Instructor emphasised that preserving food allows you to enjoy garden produce year-round and avoid wasting garden produce		
Outcome expectations/positive self-evaluation	Instructor emphasised that frozen vegetables are just as nutritious as fresh and take less time to prepare	Instructor emphasised that canning is an activity to do with family or friends, canned goods make unique gifts that others enjoy (social outcome expectations)	
Skills and knowledge	Instructor presented steps of freezing various fruits/vegetables and provided easily customisable recipes for frozen vegetables	Instructor did a live demonstration of filling canning jars Instructor showed a video clip of the respective canning method Instructor showed examples of canning equipment and supplies, explaining what each item is used for The class read a canning recipe together	
Self-efficacy	Instructor emphasised that experimenting with freezing fruits and vegetables is low risk	Instructor emphasised that even if canning did not go smoothly, the final product is still edible and provided an opportunity to practice Instructor emphasised that just like gardening, any new skill takes practice Instructor emphasised starting slow Instructor emphasised that the risk of botulism is extremely low	

The freezing lesson described how to freeze your garden fruits and vegetables to maintain the best quality. The cooking skills needed for freezing are substantially less complicated than canning, therefore this lesson also focused heavily on how to use your frozen produce. Participants were provided with basic, easily adaptable recipes to make healthy dishes from their frozen vegetables.

The water bath canning lesson and the pressure canning lesson both covered the basic steps of the particular canning method. These lessons emphasised the importance of using tested recipes and provided several options of places to find tested recipes. Demonstrations and videos were also included. For example, the instructor showed examples of canning equipment and supplies and described what they are used for, and then the instructor demonstrated how to fill a jar for canning. Video clips of portions of the canning process were also shown to approximate a live demonstration. Participants were also provided with handouts to take home. One included safe recipe substitutions and the other provided a one page ‘cheat sheet’ with the basic steps of water bath canning or pressure canning that participants can use during the canning process.

### Step N: nail down evaluation

The final step in the DESIGN process is ‘Nail Down the Evaluation.’ For step N, we developed an outcome evaluation plan that consisted of post-lesson and follow-up surveys. Table 3 contains a selection of the post-lesson evaluation questions, and Table 4 contains a selection of the follow-up survey questions. The post-lesson evaluation included questions that assessed the educational objectives created in step I. Post-lesson surveys were administered immediately following each lesson, and the questions were adapted from other food preservation evaluations or developed by the investigators.

**Table 3:** Results from step N (nail down the evaluation): selected post-lesson evaluation questions

Post-lesson Evaluation: Questions to assess educational objectives	
Educational Objective	Questions
Increase confidence in the ability to safely preserve food	‘*Before* this lesson, how confident were you in (freezing/water bath canning/pressure canning) your garden produce?’* ‘*After* this lesson, how confident are you in (freezing/water bath canning/pressure canning) your garden produce?’*
Decrease food safety fears of home canning	*Before* this lesson, how worried were you about food safety with home (water bath canning/pressure canning)?† *After* this lesson, how worried are you about food safety with home (water bath canning/pressure canning)?†
Know safe canning or freezing practices	3–4 true or false knowledge questions for each lesson. Example questions: Freezing lesson: ‘You should wash produce before freezing it, even if you garden organically.’ Water bath canning lesson: ‘It is safe to process jars for less time than the recipe says.’ Pressure canning lesson, ‘Processing time starts once you reach the pressure given in the recipe.’

* Response options were on a five-point scale, from 1 = not at all confident to 5 = extremely confident.

† Response options were on a five-point scale, from 1 = not at all worried to 5 = extremely worried.

**Table 4:** Results from step N (nail down the evaluation): selected follow-up survey evaluation questions

Question topic	Question(s)
Assess barriers and challenges to preservation	Did you have any challenges with (freezing food/water bath canning/pressure canning), or did anything keep you from (freezing/canning) as much as you wanted? If yes, please explain.
Assess utility of lessons for preserving food	Could anything from the food preservation lesson(s) you attended be changed or added that would have helped you with preserving food? Please explain. What parts of the food preservation class/classes you attended were the most useful when you were preserving? Could the food preservation class have provided any other information or resources or information to help you during the preservation process? If yes, please explain.
Assess perceived benefits of preserving food	What did you like about preserving food? (open-ended) Because I preserved food…* I eat more vegetables. I eat more fruit. I spend less money on food. I am better able to provide food for my family and myself. I feel better about where my food comes from. I wasted less food.

* Participants indicated their agreement with each statement on a four point scale with the following answer choices: Strongly Agree, Agree, Disagree, Strongly Disagree. These questions were adapted from the Community Food Projects Evaluation Toolkit.

The follow-up survey examined class attendees’ experiences with preserving, challenges and barriers to preserving and perceived benefits of preserving food. Follow-up survey questions were adapted from the Community Food Project Evaluation Toolkit or developed by the investigators^(33)^. Participants provided informed consent prior to completing evaluation surveys. The follow-up surveys were designed to: (1) determine if participants preserved food at home following the lessons, (2) identify challenges participants experienced with home food preservation, (3) examine barriers that prevented participants from preserving food, (4) to assess the perceived influence of home food preservation on vegetable intake and aspects of food security, as well as other perceived benefits of home food preservation and (5) gain additional participant feedback on the lessons.

Participants were contacted in November of 2021 to complete a follow-up survey, approximately 2–3 months after the lessons. This time was selected in order to provide ample time for participants to try preserving food at home after the lessons. To incentivise completion of the follow-up survey, participants were provided with a $15 gift card. All participants who included their contact information on the post-lesson evaluation were contacted, and reminder emails about the survey were sent twice. Participants provided informed consent prior to completing the surveys. Lesson participants were free to not complete the evaluation or not answer any question(s) they were uncomfortable with. Participants who completed the post-lesson evaluations were entered into a drawing to receive a canning cookbook or set of freezer containers.

### Data analysis

To assess the impact of the lessons on participants’ confidence in preserving food, participants were asked on the post-lesson evaluation to retrospectively indicate their level of confidence before the lesson, *i.e. ‘Before this lesson, how confident were you in…’*, and to indicate their level of confidence after the lesson, i.e. ‘*After* this lesson, how confident are you in…’. A similar approach was used to assess the impact of the lessons on worry about food safety. For the two canning lessons, participants were asked on the post-lesson evaluation to retrospectively indicate their level of worry about food safety with home canning before the lesson and then indicate their level of worry after the lesson. To assess if worry or confidence significantly differed pre- and post-lesson, Wilcoxon signed-rank tests were used. All other analyses on closed-ended questions were descriptive (i.e. n and percent). Responses to the open-ended questions were summarised.

## Results

Despite substantial outreach efforts (social media posts, inclusion on email newsletters and appearing on a local news segment), class attendance was lower than planned. The COVID-19 pandemic likely contributed to this. We aimed for 40 participants for each type of food preservation (20 per class, two classes per type of food preservation). In total, 14 people completed the freezing evaluation (86 % female, mean age 59 years), 11 people completed the water bath canning evaluation (64 % female, mean age 43 years) and six people completed the pressure canning evaluation (83 % female, mean age 52 years).

**Fig. 1:**
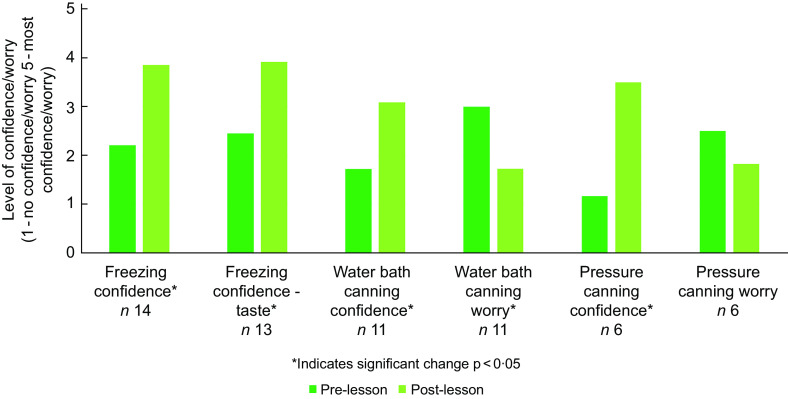
Mean food preservation confidence and worry scores pre-lesson and post-lesson. Pre-lesson scores were assessed retrospectively after each lesson

The post-lesson evaluations included three to four questions to measure the effectiveness of this curriculum at imparting knowledge of safe food preservation practices. For the freezing lesson, 11 out of 13 respondents answered all the knowledge questions correctly, 8 out of 11 water bath canning lesson attendees answered all questions correctly and 5 out of 6 pressure canning attendees answered all the knowledge questions correctly. Results for the confidence and worry questions are found in Fig. 1. Confidence for each of the three types of food preservation significantly increased, as did confidence in making good-tasting food from frozen vegetables. Worry about food safety with water bath canning decreased, but worry about food safety with pressure canning did not significantly change.

### Follow-up survey results

Out of the 15 respondents who completed the follow-up survey, 87 % were female and the mean age was 53 years. Fourteen reported preserving food after the lesson: nine reported freezing, eight reported water bath canning and two reported pressure canning. Only one participant reported not preserving any food. With regard to food preservation challenges and barriers, only one person reported a challenge or barrier to freezing their produce: that the produce went bad before they froze it. Barriers to water bath canning included lack of time and concern over food safety. For example, one participant said: ‘I got overwhelmed after the class that I wouldn’t get a good seal or wouldn’t get a proper top space and might unknowingly breed bacteria.’ Regarding challenges experienced with water bath canning, one participant stated, ‘My biggest frustration is not being able to customize recipes. The final product seemed mild compared to specialty products available,’ while another said, ‘I had to use an outdoor burner for it because I have an electric stove. This is keeping me from doing this more often.’ Other challenges during water bath canning included seal failure, incorrectly following the recipe and difficulty finding jar lids. For pressure canning, time was reported as a barrier, and no challenges were reported.

The follow-up surveys offered an opportunity to obtain feedback on the food preservation lessons after participants had tried food preservation at home. Overall, participants found the information from the lessons helpful and liked the handouts and links to additional resources that were provided. One participant enjoyed that the curriculum taught the ‘Importance of following tried and true recipes and how to substitute.’ Another participant stated they liked, ‘the demonstrations and the reassurance that botulism poisoning is extremely rare in the US from home canning.’ When asked how to improve the lessons, several attendees of the water bath or pressure canning class wanted live canning demonstrations and/or being able to do hands-on canning in class. One participant said ‘I wish I had the confidence to try canning. I would like it if I could have …a class where we actually can something, even if it’s one thing!’

Participants who reported preserving food (*n* 14) were also asked about the perceived influence of home food preservation on vegetable intake and aspects of food security. Nearly two-thirds of participants (64 %) agreed or strongly agreed that they ate more vegetables because of preserving food, and the same results were found for fruit. For the questions assessing aspects of food security, 57 % of respondents agreed or strongly agreed that they spend less money on food due to preserving, while 71 % reported being better able to provide food for themselves and their family. Lastly, 93 % reported feeling better about where their food comes from and wasting less food due to preserving. From the open-ended question asking participants ‘what did you like about preserving food?’, the most common theme was related to enjoying eating their garden food throughout the winter, with one participant saying, ‘I like having a jar of summer sunshine to open and enjoy in the middle of winter.’ Others reported liking that they used more of their garden produce, self-sufficiency and liking the taste of garden the home-preserved food.

## Discussion

In this study, we describe how a program planning framework, the DESIGN process, was used to develop food preservation lessons for gardeners. These lessons built on the preexisting gold standard sources of tested food preservation information, the National Center for Home Food Preservation and the USDA Complete Guide to Home Canning^(28,29)^ and incorporated social cognitive theory to address determinants of behaviour change. Our post-lesson evaluation of the lessons examined participants’ knowledge of proper food preservation practices and confidence in home food preservation. A follow-up survey was also conducted to identify challenges participants experienced with home food preservation and assess the perceived influence of home food preservation on fruit and vegetable intake and aspects of food security.

One strength of the DESIGN process is its focus on incorporating determinants of behaviour change beyond just nutrition knowledge. According to Contento and Koch, ‘Knowledge or nutrition literacy is not enough’ to ensure a healthy diet^(14)^. In fact, a systematic review found that while nutrition knowledge is significantly associated with better diet quality, the association is weak^(34)^. Many other psychosocial determinants are related to diet quality, such as self-efficacy, social support and food preferences^(35–37)^. Nutrition education that focuses on providing knowledge alone is likely insufficient to result in substantial dietary behaviour change, and incorporating other dietary behaviour determinants is needed. Our curricula incorporated several determinants beyond nutrition knowledge. We placed a special emphasis on self-efficacy since food preservation, especially canning, is a relatively complex culinary task and thus achieving high self-efficacy for preserving food may be especially challenging. In our post-lesson evaluations, food preservation confidence increased following the lessons and worry decreased, indicating an improvement in self-efficacy.

In our follow-up surveys, participants reported the perceived influence of food preservation on their diets. Most participants reported that they agreed or strongly agreed that they ate more fruit and vegetables, spent less money on food, felt better about where their food comes from, wasted less food and were better able to provide food for themselves and their family. While there is scant literature examining the dietary impacts of home food preservation, these findings are in line with the existing research on gardening. Numerous studies have found that gardeners prefer their garden produce over store-bought, value knowing how it is grown and have pride in the food they grew themselves^(11,18,19)^. The positive relationship that gardeners have with the food they grow can be extended beyond the gardening season by preserving food.

This study of the design and evaluation of food preservation lessons for gardeners was unique in several ways. First, while there are many food preservation curricula, there are very few published reports on their development or evaluation. This study is also unique in its account of programme development using a formalised planning framework, the DESIGN process. Only a few other studies examine the use of the DESIGN process for developing curricula, and none of these curricula have a focus on food safety topics^(38–40)^. The most prominent weakness of this study is the small number of participants who completed the post-lesson and follow-up evaluations. While this study provides evidence that home food preservation may be beneficial in promoting fruit and vegetable intake and food security among gardeners, future research with larger sample sizes is needed.
